# Metformin therapy before conception versus throughout the pregnancy and risk of gestational diabetes mellitus in women with polycystic ovary syndrome: a systemic review, meta-analysis and meta-regression

**DOI:** 10.1186/s13098-019-0453-7

**Published:** 2019-07-23

**Authors:** Razieh Bidhendi Yarandi, Samira Behboudi-Gandevani, Mina Amiri, Fahimeh Ramezani Tehrani

**Affiliations:** 1 Reproductive Endocrinology Research Center, Research Institute for Endocrine Sciences, Shahid Beheshti University of Medical Sciences, No 24, Parvane Street, Yaman Street, Velenjak, P.O.Box: 19395-4763, Tehran, Iran; 2 Department of Epidemiology and Biostatistics, School of Public Health, Tehran University of Medical Sciences, Tehran, Iran

**Keywords:** Gestational diabetes mellitus, Meta-analysis, Meta-regression, Metformin therapy, Polycystic ovary syndrome

## Abstract

The results of studies that assessed the impact of metformin treatments on gestational diabetes mellitus (GDM) in patients with polycystic ovary syndrome (PCOS) are inconclusive. In addition, the impact of time and duration of metformin therapy for an optimum reduction of GDM has not been reported in these studies. This study aimed to summarize current knowledge regarding the effect of metformin-therapy before conception versus throughout pregnancy on the risk of GDM in women with PCOS. PubMed, Scopus, Google Scholar and ScienceDirect databases were searched to identify relevant studies. Both fixed and random effect models were used. Subgroup analyses were performed based on the on the study methodology. The association between the PCOS status and GDM was assessed using the univariate and multiple meta-regression analysis adjusted by the BMI and metformin therapy. Forty-eight of 1397 identified studies were included involving 5711 PCOS patients and 20,296 controls. Regardless of metformin therapy, the prevalence of GDM diagnosed in the second trimester among women with PCOS was significantly higher than healthy controls that was independent of obesity. Including all studies, the increased risk of GDM among women with PCOS, compared to healthy controls, disappeared after the adjustment of metformin-therapy (β = 0.08, 95% CI 0.04, 0.2; p = 0.624). By excluding observational studies as a source of bias, the prevalence of GDM among women with PCOS treated using metformin before conception till the end of pregnancy did not differ from treated just before conception (β = − 0.09, 95% CI − 0.2, 0.02; p = 0.092) or those without metformin therapy (β = − 0.05, 95% CI − 0.07, 0.04; p = 0.301). The results remained unchanged after the subgroup analysis based on the methodology of RCTs and non-RCTs studies. The main body of literature in the current meta-analysis was observational, which may be mixed with some sources of bias. Also, a lack of well-designed and high quality interventional studies means that the findings should be interpreted with cautious. In this respect, decisions regarding the continuation or discontinuation of metformin therapy in women with PCOS are somewhat arbitrary and can be made individually based on the patient’s condition given the presence or absence of other GDM risk factors. Additional well-designed RCTs still need for precise recommendation.

**Electronic supplementary material:**

The online version of this article (10.1186/s13098-019-0453-7) contains supplementary material, which is available to authorized users.

## Background

Polycystic ovary syndrome (PCOS) with a prevalence of 7–15% is one of the most common endocrinopathies among women in the reproductive age [1]. Hyperandrogenism and/or hyperandrogenemia, chronic oligo-ovulation and polycystic ovaries morphology are the main characteristics of this syndrome. The exact underlying pathogenic mechanisms of PCOS are not fully understood, but it is believed that insulin resistance (IR) with compensatory hyperinsulinemia is the cornerstone of its pathogenesis [2, 3].

It is well documented that non-pregnant women with PCOS face more metabolic and reproductive complications with an early or late term syndrome’s risks [4–6]. However, the effects of PCOS on pregnancy outcomes remain controversial. Normal pregnancy is characterized by the physiologic insulin resistance state, which is at its peak in the third trimester of pregnancy. Human placental lactogen, estradiol, progesterone and cortisol regulate the insulin status during pregnancy, which induce the diabetogenis state due to the facilitated diffusion and transfer of glucose to the fetus [7–9]. Pregnant women suffering from PCOS experience the additive preexisting state of insulin resistance, which may accompany adverse pregnancy outcomes [10]. Metformin as an insulin sensitizing agent have been wildly used for PCOS, but its effect on the prevention of GDM in PCOS is controversial.

According to available meta-analyses studies, women with PCOS have 2.8–4.3 higher risk of GDM compared to healthy controls [5, 10–13]. Mopreover, several studies were conducted to assess the impact of metformin treatments on GDM in patients with PCOS [14–19]. However, their results were inconclusive. For instance, Zheng et al. [19] in a meta-analysis study showed that the incidence of GDM was significantly lower among pregnant women with PCOS receiving metformin than those not received. Conversely, according to another meta-analysis, Zhuo et al. [17], metformin did not significantly reduced GDM in women with PCOS. These controversial results may be partly explained by the use of different eligibility criteria for the type of included studies (interventional versus observational) or selecting a non-homogenous control groups (PCOS not treated, or both not treated PCOS and non-PCOS ones) [14–17, 19], and not adjustment for most relevant confounders including age and body mass index. Moreover [18, 19], most of those meta-analyses did not assess the quality of included studies [14, 15, 18, 19] and none of them evaluated the risk of bias [14–19]. In addition, the impact of time and duration of metformin therapy for an optimum reduction of GDM has not been reported in these studies. Hence, we decided to conduct a meta-analysis to assess the effect of metformin-therapy before conception versus all throughout the pregnancy on the risk of gestational diabetes mellitus (GDM) in women with polycystic ovary syndrome (PCOS) after the adjustment for type of study (observational versus trials), age and BMI.

## Methods

This systematic review and meta-analysis was conducted based on the Preferred Reporting Items for Systematic Reviews and Meta-Analyses (PRISMA) statement [20] with the following objectives:

- Study of the prevalence of GDM among women with PCOS regardless of metformin therapy, compared to healthy controls;
- Study of the effect of obesity on the prevalence of GDM among women with PCOS, compared to healthy controls;
- Study of the prevalence of GDM among women with PCOS treated with metformin just before conception/before conception till the end of pregnancy, compared to healthy controls;
- Study of the prevalence of GDM among women with PCOS treated with metformin before conception until the end of pregnancy, compared to women with PCOS treated with metformin just before conception.
- Study of the prevalence of GDM among women with PCOS treated with metformin only before conception/before conception until the end of pregnancy, compared to untreated women with PCOS.

### Search strategy, study selection and data extraction

A comprehensive literature search was performed in the PubMed (including Medline), Web of Science and Scopus databases for retrieving relevant randomized or non-randomized controlled trials (RCTs or NRS), cohort studies, cross sectional, and case–control studies published in English language up to August 2017. In addition, a manual search of the reference list of relevant studies was conducted to expand the search coverage.

The following MeSH terms keywords, alone or in combination, were used for the search process: “Polycystic Ovary Syndrome” OR “Polycystic Ovarian Syndrome” OR “polycystic ovary disease” OR “PCOS” OR “PCOD” OR “Stein Leventhal Syndrome” AND “insulin resistance” OR “gestational diabetes” OR “pregnancy complications” OR “obstetric complications” OR “adverse pregnancy outcome”.

The initial selection of articles was performed based on titles’ screening, followed by a second round of selection performed by one reviewer, who deleted duplicates and reviewed the abstracts of all remaining records. Any disagreement in the selection of abstracts was resolved through consensus or by a senior reviewer. The full text articles were evaluated.

Studies with subjects having diabetes or currently using antidiabetic drugs except metformin, reporting the prevalence of PCOS retrospectively in women with GDM and non-original studies were excluded. General characteristics of the studies including “authors, journal, publication year, design, recruitment source, ethnicity, sample size for cases and controls as well as group characteristics including diagnostic criteria of PCOS, screening time and strategy of GDM, age, body mass index (BMI), duration of metformin therapy, adjustment methods of confounders and prevalence of GDM were extracted.

### Quality assessment

Quality of the studies was critically apprised in terms of methods and results. Two reviewers who were blind to the study’s author, journal and institution evaluated quality of the studies independently. Disagreements were resolved through consensus or by a senior reviewer.

The modified Consolidated Standards of Reporting Trials (CONSORT) was used as a validated quality assessment checklist for clinical trials [21]. Studies with a score ≥ 70% of the highest level of the CONSORT checklist score were considered as high quality, those with 40–70% of the score as moderate, and those with 20–40% of the score as low quality and with < 20% of the score as very low quality.

The quality of observational studies was also evaluated using the modification of the Newcastle–Ottawa Quality Assessment Scale for Nonrandomized Studies (NRS) [22], which assessed the quality of published nonrandomized studies in terms of selection, comparability and outcome. Studies with a score above 6 were considered high quality, 3–5 moderate and below than 3 low quality.

### Risk of bias assessment

The risk of bias of NRS and other methodological studies was assessed using the ROBINS [23] and Cochrane Collaboration’s tool, respectively [24]. In this respect, the risk of bias based on the subgroups of low-, moderate-, critical- and unclear risk was assessed.

### Statistical analysis

The STATA software package (version 12; STATA Inc., College Station, TX, USA) was used to conduct statistical analysis. Heterogeneity was evaluated using the Chi square test and P value > 0.05 was interpreted as homogeneity. Publication bias was assessed using the Begg’s test as a formalized statistical test for statistically estimating funnel plot asymmetry to find any possible publication bias. Accordingly, the random effect model without any correction were used for such analysis. The Meta-prop method was used for the pooled estimation of the prevalence of GDM. The Mantel–Haenszel method for meta-analysis was applied for the pooled estimation of age and BMI in various subgroups including women with PCOS (without metformin therapy, metformin therapy just before conception, and metformin therapy before conception until the end of pregnancy) and non-PCOS women. In addition, subgroup analysis was performed based on the study methodology. The association between the PCOS status and GDM was assessed using the univariate and multiple meta-regression analysis adjusted by the BMI and metformin therapy. The prevalence of GDM, PCOS status and weight given to each study was calculated using the fixed effect model based on the inverse of within-study variance, and was presented through the scatter bubble plots. P > 0.05 was set as statistically significant.

## Results

### Search and study selection

The search yielded 1397 potentially relevant articles. The flow chart indicating the selection process for the systematic review and meta-analysis was depicted as Additional file 1: Fig. S1. According to the inclusion criteria, 48 full-text articles were selected for the meta-analysis.

### Study characteristics

Forty-eight studies published between 1998 and 2017 were included in the systematic review. Data on 5711 women with PCOS and 20,296 healthy controls was presented in Table 1. Overall, most studies were judged as having a low risk of bias for evaluated domains (Additional file 1: Figs. S2–S5). In addition, quality of the body of evidence in the current meta-analysis was classified as moderate. Twenty-three studies were identified as high quality [25–47] and other as moderate quality (Additional file 1: Tables S1–S3).

**Table 1:** Summary of studies assessing GDM prevalence in women PCOS and controls with or without metformin therapy

Author, year	PCOS criteria	Time screening, Guideline	Group 1 characteristics (PCOS patient with metformin therapy in pregnancy)	Group 2 characteristics (PCOS patient without metformin therapy in pregnancy)	Group 3: characteristics (Non-PCOS pregnant group)	Metformin therapy in PCOS	Prevalence of GDM (%)
Abd El Hameed et al. (2011)^1^	Rotterdam	Time: 8,24,36 w, guideline: NM°	N = 31, Age: 30.2 (3.8), BMI: 29.22 (2.3)	N = 26, Age: 28.1 (4.3), BMI: 28.3 (1.9)	–	Group 1: before conception till the end of pregnancy Group 2: –	2ed trimester Group 1: 3.2 Group 2: 23.08
Begum et al. (2009)^1^	Rotterdam	Time: NM guideline: NM	N = 29, Age: 28.1 (2.9), BMI: 28.2 (2.3)	N = 30, Age: 26.1 (3.6), BMI: 27.9 (2.4)	–	Group 1: before conception till the end of pregnancy Group 2: before conception	Group 1: 3.44 Group 2: 30
Ashrafi et al. (2014)^2^	Rotterdam	Time: 24–28 w, guideline: ADA	–	N = 234, Age: 29.6 (3.9), BMI: (26.1)	Group 3a: non-PCOS, infertile N = 234, Age: 30.7 (4.7), BMI: 25.5 (4.2) Group 3b: non-PCOS, fertile N = 234, Age: 26.4 (5.5), BMI: 25.7 (3.8)	Group 2: before conception Group 3: – Group 4: –	Group 1: 44.4 Group 3a: 29.9 Group 3b: 7.3
Ashrafi et al. (2017)^2^	Rotterdam	Time: 24–28 w, guideline: ADA	–	Group 2a: HA + AO + PCO N = 113, Age: 29.5 (3.8), BMI: 26.1 (3.1) Group 2b: AO + HA N = 5, Age: 28.6 (5.4), BMI: 27.7 (3.2) Group 2c: HA + PCO N = 74, Age: 29.90 (4.2), BMI: 25.94 (4.1) Group 2d: AO + PCO N = 16, Age: 28.3 (2.8), BMI: 25.9 (2.1)	–	Group 2a: no Group 2b: no Group 2c: no Group 2d: no	Group 2a: 46 Group 2b: 100 Group 2c: 41.9 Group 2d: 43.8
Bjercke et al. (2002)^3^	NIH	TIME: NM, guideline: NM	–	Group 2 a: without IR N = 29, Age: 31.5 (3.8), BMI: 25.2 (3.9) Group 2 b: with IR N = 23, Age: 31.1 (4.0), BMI: 27.7 (5.5)	N = 355, Age: 32.7 (3.4), BMI: 21.9 (2.7)	–	Group 2a: 7 Group 2b: 9 Group 3: 0.6
D’Anna et al. (2012)^3^	NM	Time: 24–28 w, guideline: NM	–	N = 37, Age: 30.6 (4.2), BMI: 24.7 (3.9)	–	Group 2: before conception	Group 2: 54
De Fre`ne et al. (2014)^3^	Rotterdam	Time: ~ 24 w, guideline: ADA	–	Group 2 a: overweight N = 93, Age: 29 (4.2), BMI: 30.8 (27.7–33.5)^a^ Group 2 b: Normal weight N = 107, Age: 28.4 (3.1), BMI: 20.9 (20–22.3)^a^	–	Group 2 a: NO Group 2 b: NO	Group 2 a: 8.2 Group 2 b: 0
De Leo et al. (2011)^3^	AES	Time: NM, guideline: NM	N = 98, Age: 32 (6), BMI: 28.3 (2.1)	–	N = 110, Age: 33 (5), BMI: 26.6 (1.2)	Group 1: before conception till 37 weeks’ gestation Group 2:–	Group 1: 0 Group 3: 12.5
deWilde et al. (2015)^3^	Rotterdam	Time: 24–26 w, guideline: ADA	–	N = 72, Age: 29.6 [26.8–31.8]^a^, BMI: 24.4 [21.6–28.9]^a^	–	Group 2: before conception	Group 2: 31
deWilde et al. (2014)^3^	Rotterdam	Time: 24–26 w, guideline: NM	–	N = 189, Age: 29 [27–31]^a^, BMI: 24 [21–28]^a^	–	Group 2: before conception	Group 2: 22
Dmitrovic et al. (2011)^2^	NIH	Time: 6–10, 12–16, 24–28, 34–38 w, guideline: ADA	–	N = 17, Age: 29 (4), BMI: 32 (8)	N = 17, Age: 31 (5), BMI: 26 (7)	Group 2: – Group 3: –	Group 2: 47 Group 3: 12
Elkholi et al. (2016)^2^	Rotterdam	Time: 24–28 w, guideline: ADA	–	Group 2a: metabolically obese N: 62, Age: 22.3 (1.2), BMI: 21.4 (1.5) Group 2b: metabolically healthy N: 47, Age: 21.1 (1.6), BMI: 21.6 (1.4)	N: 35, Age: 20.4 (1.3), BMI: 21.3 (1.4)	Group 2a: before conception Group 2b: before conception Group 3: No	Group 2a: 9.8 Group 2b: 0 Group 3: 0
Fougner et al. (2008)^1^	Rotterdam	Time: 19, 32, 36 w, guideline: WHO	N = 18, Age: 28.9 (26.5–31.4)^a^, BMI: 32.1 (29.1–35.2)^a^	N: 22, Age: 28.3 (26.6–30.0)^a^, BMI: 29.3 (25.8–32.9)^a^	–	Group 1: before conception till the end of pregnancy Group 2: before conception	First trimester Group 1: 11.1 Group 2: 27.2 2ed trimester Group 1: 11.1 Group 2: 4.5 3rd trimester Group 1: 22.2 Group 2: 9
Glueck et al. (2004)^3^	Rotterdam	Time: 26–28 w, guideline: ADA	N = 90, Age: 33 (5), BMI: 33.8 (7.8)	–	N = 252, Age: 29 (6), BMI: 25.6 (5.9)	Group 1: before conception till the end of pregnancy Group 3: –	Group 1: 9.5 Group 3: 15.9
Glueck et al. (2004)^3^	Rotterdam	Time: 26–28 w, guideline: ADA	N = 39, Age: 30 (4), BMI: 34 (8.2)	–	–	Group 1: before conception till the end of pregnancy	Group 1: 7.6
Glueck et al. (2002)^3^	NIH	Time: 26–28 w, guideline: ADA	N = 33, Age: 34 (8), BMI: 33.9	–	–	Group 1: before conception till the end of pregnancy	Group 1: 33
Glueck et al. (2013)^3^	Rotterdam	Time: NM, guideline: NM	N = 76, Age: 32 (5), BMI: 33.3 (7.4)	–	N = 156, Age: 30 (6), BMI: 26.9 (6.6)	Group 1: before conception till the end of pregnancy Group 3:–	Group 1: 10.5 Group 3: 14.7
Glueck et al. (2008)^3^	Rotterdam	Time: 26–28 w, guideline: ADA	N = 142, Age: 30 (5), BMI: 33.5 (7.9)	–	–	Group 1: before conception till the end of pregnancy	Group 1: 7
Glueck et al. (2002)^3^	Rotterdam	Time: 26–28 w, guideline: ADA	–	N = 68, Age: –, BMI: 33 (29–38.8)^a^	–	Group 2: before conception	Group 2:
Haakova et al. (2003)^3^	Rotterdam	Time: second, third trimester, guideline: NM	–	N = 66, Age: 29.8 (4.9), BMI: 23.2 (3.8)	N = 66, Age: 29 (4.9), BMI: 23.2 (3.8)	Group 2: – Group 3: –	Group 2: 4.92 Group 3: 12.12
Han et al. (2011)^3^	Rotterdam	Time: 24 w, guideline: ADA	–	Group 2a: Obese N = 64, Age: 31.6 (3.1), BMI: 27.46 (2.4) Group 2b: Non-obese N = 272, Age: 31.2 (2.7), BMI: 20.45 (2.0)	Group 3a: Obese N = 117, Age: 32.2 (3.2), BMI: 27.5 (2) Group 3b: Non-obese N = 886, Age: 32.5 (2.8), BMI: 20.5 (1.9)	Group 2a: no Group 2b: no Group 3a: no Group 3b: no	Group 2a: 10.5 Group 2b: 1.1 Group 3a: 8.6 Group 3b: 1.8
Hassanzahraeiet al. (2007)^3^	NIH	Time: 24–28 w, guideline: NDDG	–	N = 47, Age: 27.8 (5.2), BMI: 25.1 (4.4)	N = 100, Age: 28 (4.9), BMI: 23.4 (3.3)	Group 2: – Group 3: –	Group 2: – Group 3: –
Joham et al. (2014)^2^	NM	Time: NM, guideline: NM	–	N = 478, Age: 30.5 (1.4), BMI: 28 (7.2)	N = 8134, Age: 30.6 (1.5), BMI: 25.1 (5.6)	Group 2: – Group 3: –	Group 2: 11.2 Group 3: 3.8
Khattab et al. (2011)^3^	Rotterdam	Time: 5–12, 19, 32, 36 w, guideline: WHO	N = 31, Age: 30.2 (3.8), BMI: 29.22 (2.3)	N = 31, Age: 30.2 (3.8), BMI: 29.22 (2.3)	–	Group 1: before conception till the end of pregnancy Group 2: before conception	Group 1: 4 Group 2: 20
Kollmann et al. (2015)^3^	1-NIH 2. Rotterdam (HA + PCO) 3. Rotterdam (OA + PCO)	Time: 24–28 w, guideline: IADPSG	–	Group 2a: NIH criteria N = 85, Age: 29 (26–32)^a^, BMI: 24.3 (21.4–29.2)^a^ Group 2b: Rotterdam (HA + PCO) N = 14, Age: 31 (26–33) ^a^, BMI: 25.5 (22.1–31.2)^a^ Group 2c: Rotterdam (OA + PCO) N = 78, Age: 30 (27–33)^a^, BMI: 24.2 (20.5–29.7)^a^	N = 708,Age: 30 (25–34)^a^, BMI: 22.5 (20.5–25.8)^a^	Group 2a: – Group 2b: – Group 2c: –	Group 2a: 18.8 Group 2b: 14.3 Group 2c: 26.9 Group 3: 2.5
Lesser et al. (1997)^3^	NIH	Time: 20–28 w, guideline: NDDG	–	N = 24, Age: 29.8 (5.3), BMI: 28.4 (4.7)	N = 44, Age: 32 (4.6), BMI: 23.4 (2.79)	Group 2: no Group 3: no	Group 2: 16.7 Group 3: 6.7
Mehrabian et al. (2013)^3^	Rotterdam	Time: 24–28 w, guideline: ADA	–	Group 2a: PCOS with GDM N = 50, Age: 34 (47.5), BMI: 28.9 (4.5) Group 2a: PCOS without GDM N = 130, Age: 33.3 (6.6), BMI: 25.9 (4.5)	–	Group 2: no	Group 2 totally: 27.8
Mikola et al. (2001)^3^	NIH	Time: NM, guideline: NM	–	N = 99, Age: 30.4 (3.9), BMI: 25.6 (6.5)	N = 737, Age: 29.4 (4.8), BMI: 23 (4.6)	Group 2: no Group 3: no	Group 2: 20 Group 3: 9
Mumm et al. (2015)^3^	Rotterdam	Time: 14–20, 28–30 w guideline: NM	–	N = 157, Age: 29 (26–32)^a^, BMI: 25.9 (22.0–32.0)^a^	N = 995, Age: 29 (26–33)^a^, BMI: 23.2 (20.9–26.1)^a^	Group 2: no Group 3: no	Group 2: 6.4 Group 3: 13.8
Naver et al. (2014)^3^	Rotterdam	Time: NM, guidelines: national	–	N = 459, Age: 31.6, BMI: 22.9	N = 5409, Age: 30.7, BMI: 23.4	Group 2: no Group 3: no	Group 2: 2.4 Group 3: 1.1
Nawaz et al. (2008)^3^	Rotterdam	Time: 24–28 w, guideline: NM	Group 1a: N = 40, Age: 28 (3.6), BMI: 29.6 (5.1) Group 1b: N = 20, Age: 29 (3.1), BMI: 30 (2.6) Group 1c: N = 45, Age: 27 (4.2), BMI: 29.3 (3.3)	N = 32, Age: 30 (2.9), BMI: 31.2 (4.6)	–	Group 1a: before conception till 4–16 weeks of gestation Group 1b: before conception till 32 weeks of gestation Group 1c: before conception till the end of pregnancy Group 2: no	Group 1a: 37.5 Group 1b: 50 Group 1c: 28.8 Group 2: 40.6
Ott et al. (2014)^3^	Rotterdam	Time: second trimester, guideline: NM	–	Group 2a: conceived with LOA + metformin N = 40, Age: 27.8 (4.9), BMI: 26.9 (5.0) Group 2b: conceived with CC + metformin N = 40, Age: 27.5 (4.5), BMI: 28.0 (6.0) Group 2c: conceived with metformin only N = 40, Age: 27.2 (4.6), BMI: (27.2 ± 5.6)	–	Group 2a: before conception Group 2b: before conception Group 2c: before conception	Group 2a: 29.4 Group 2b: 31.3 Group 2c: 31.4
Palomba et al. (2010)^3^	Rotterdam	Time: NM, guideline: NM	–	N = 93, Age: 30 (20–33)^a^, BMI: 24.2 (18.1–29.1)^a^	N = 73, Age: 30 (19–34)^a^, BMI: 24 (17.8–29.4)^a^	Group 2: no Group 3: no	Group 2: 16.1 Group 3: 5.8
Paradisi et al. (1998)^3^	NIH	Time: NM, guideline: NM	–	Grou2a: PCOS with GDM N = 5, Age: 32.2 (6.3), BMI: 28.3 (0.7) Group 2b: PCOS without GDM N = 8, Age: 28 (2.9), BMI: 28.3 (3.2)	–	Group 2: no	Group 2: 38.4
Radon et al. (1999)^3^	ICD-9th revision	Time: 24–28 w, guideline: NM	–	N = 22, Age: 32.4 (4.1), BMI: 28.9 (8)	N = 66, Age: 31.1 (3.9), BMI: 28 (7.2)	Group 2: no Group 3: no	Group 2: 40.9 Group 3: 3
Reyes-Muñoz et al. (2012)^3^	Rotterdam	Time: 14–24 or 24–28 w, guideline: ADA	–	N = 52, Age: 29.1 (3.9), BMI: 27.5 (3.1)	N = 26, Age: 29 (3.8), BMI: 27.5 (3.3)	Group 2: before conception Group 3: –	Group 2: yes Group 3: no
Sterling et al. (2016)^3^	Rotterdam criteria	Time: NM, guideline: NM	–	N = 71, Age: 33 (30–35)^a^, BMI: 22.7 (20.4–28.3)^a^	N = 323, Age: 35 (32–37)^a^, BMI: 22.6 (20.8–26.0)^a^	Group 2: no Group 3: no	Group 2: 15.5 Group 3: 5
Turhan et al. (2003)^3^	NIH	Time: 24–28 w, guideline: ADA	–	N = 38, Age: 27.6 (3.7), BMI: 31.5 (4.5)	N = 136, Age: 26.6 (4.7), BMI: 23.6 (4.3)	Group 2: no Group 3: no	Group 2: 2.6 Group 3: 8.1
Vollenhoven et al. (2000)^3^	NM	Time: 24–28 w, guideline: WHO	–	N = 60, Age: –, BMI: 27.1 (5.2)	N = 60, Age: –, BMI: 26.5 (4.9)	Group 2: no Group 3: no	Group 2: 22 Group 3: 17
Vanky et al. (2004)^1^	Rotterdam	Time: 19, 32, 36 w, guideline: WHO	N = 18, Age: 28.9 (4.8), BMI: 32.1 (6.1)	N = 22, Age: 28.3 (3.7), BMI: 29.3 (8)	–	Group 1: before conception till the end of pregnancy Group 2: before conception	First trimester Group 1: 16.6 Group 2: 27.2 2ed trimester Group 1: 11.1 Group 2: 4.5 3rd trimester Group 1: 22.2 Group 2: 9
Vanky et al. (2010)^1^	Rotterdam	Time: NM, guideline: NM	N = 135, Age: 29.6 (4.4), BMI: 29.5 (7)	N = 138, Age: 29.2 (4.4), BMI: 28.5 (7.2)	–	Group 1: before conception till the end of pregnancy Group 2: before conception	Group 1: 16.2 Group 2: 15.2
Vanky et al. (2011)^2^	1. NIH 2. Rotterdam	Time: 14, 28 w, guideline: WHO	–	Group 2a: PCOS based on NIH criteria N = 164, Age: 29.1 (4.4), BMI: 29.5 (6.62) Group 2a: PCOS based on Rotterdam criteria N = 93, Age: 29.6 (4.4), BMI: 27.4 (6.4)	–	Group 2a: no group 2b: no	First trimester Group 2a: 9 group 2b: 10 2ed trimester Group 2a: 11.1 Group 2b: 4.5 3rd trimester Group 1: 10 Group 2: 7
Veltman-Verhulst et al. (2010)^3^	Rotterdam	Time: 24–26 w, guideline: ADA	–	Group 2a: PCOS with GDM N = 21, Age: 26.6 (3.5), BMI: 28.2 (5.8) Group 2a: PCOS without GDM N = 29, Age: 25.6 (3.0), BMI: 24.7 (5.7)	–	Group 2: –	Group 2 totally: 42
Wan et al. (2015)^3^	Rotterdam	Time: NM, guideline: WHO	–	N = 25, Age: 31.4 (2), BMI: 22.8 (3.6)	N = 174, Age: 32.7 (3.1), BMI: 21.5 (2.6)	Group 2: no Group 3: no	Group 2: 29.2 Group 3: 29.8
Wang et al. (2013) (120)^3^	Rotterdam	Time: 24–28 w, guideline: ADA	–	N = 144, Age: 30.8 (3.9), BMI: 23 (2.6)	N = 594, Age: 29.1 (3.9), BMI: 20 (2.4)	Group 2: no Group 3: no	Group 2: 54.9 Group 3: 14.3
Weerakiet et al. (2004)^3^	NM	Time: 24–28 w, guideline: ADA	–	N = 47, Age: 31.6 (4), BMI: 24 (3)	N = 264, Age: 31.3 (3.8), BMI: 22.1 (3.6)	Group 2: no Group 3: no	Group 2: 22.2 Group 3: 18
Xia et al. (2017)^3^	Rotterdam	Time: 24–28 w, guideline: NM	–	Group 2a: PCOS with GDM N = 31, Age: –, BMI: 24.0 (6.4) Group 2a: PCOS without GDM N = 63, Age: –, BMI: 23.2 (3.0)	–	Group 2 totally: no	Group 2 totally: 32.9
Zhang et al. (2016)^2^	Rotterdam	Time: 24–28 w, guideline: ADA	–	Group 2a: PCOS with GDM N = 45, Age: 28.87 (3.20), BMI: 24.30 (3.23) Group 2a: PCOS without GDM N = 223, Age: 28.06 (3.2), BMI: 23.2 (3.1)	–	–	Group 2 totally: 16.7

*N* number, *BMI* body mass index, *NM* not mentioned, *PCOS* polycystic ovary syndrome, *HA* hyperandrogenism, *AO* anovulation, PCO polycystic ovary morphology, ADA American diabetes association, *WHO* World Health Organization

^a^Median (25th–75th percentile)

^1^Experimental study

^2^Cross sectional study

^3^Prospective study

Forty-three studies had observational and five studies had interventional (four RCTs [48–51] and one NRS [52]) methods. Marking diversity was found in screening strategies for the diagnosis of GDM. Majority of the studies performed the GDM screening test in the second trimester of pregnancy; 6 reported the GDM prevalence during the first, second and third trimesters of pregnancy [27, 49, 50, 52–54]. Twelve studies implemented the two-step screening process with a 50-g Glucose Challenge test (GCT), following a 3-h, 100-g glucose tolerance test (OGTT) [7, 24, 25, 30, 32, 38, 39, 41, 55–58]; 29 studies applied the one-step screening process with a 3-h, 100-g OGTT [28, 47, 52, 59–61] or a 2-h OGTT with 75 g glucose [27, 29, 31, 33–37, 40, 42, 44–47, 49–51, 53, 54, 62–64] and 7 studies did not mention the GDM diagnostic criteria [26, 48, 65–69]. In addition, marking diversity was found in cutoff values of diagnostic criteria (Table 1).

Twenty-seven studies did not use metformin in the women with PCOS [25–27, 29–35, 38, 40–47, 53, 61–64, 70, 71]; 13 studies used metformin therapy only before conception [25, 28, 37, 39, 48–51, 54, 55, 57, 59, 69]; 13 studies treated the women with PCOS using metformin before conception until the end of pregnancy [36, 48–52, 54, 58, 60, 65–68]; In one study, metformin therapy was used before conception until 4–16 weeks of gestations [36] and in one single study it was used before conception until the 32th week of gestation [36].

**Fig. 1:**
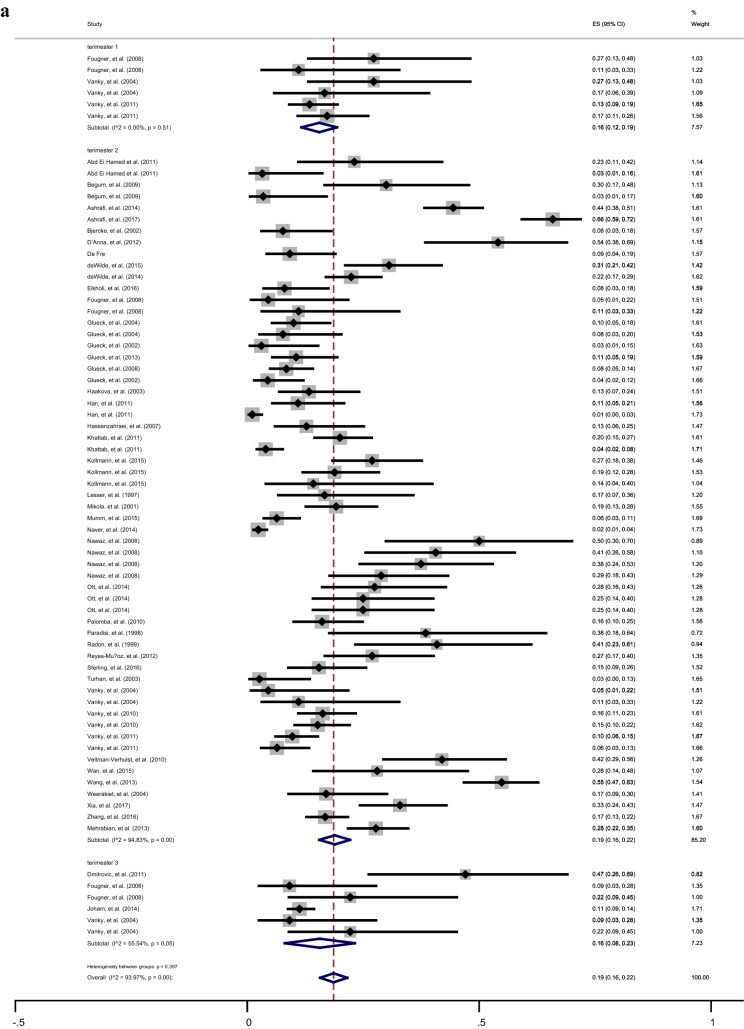

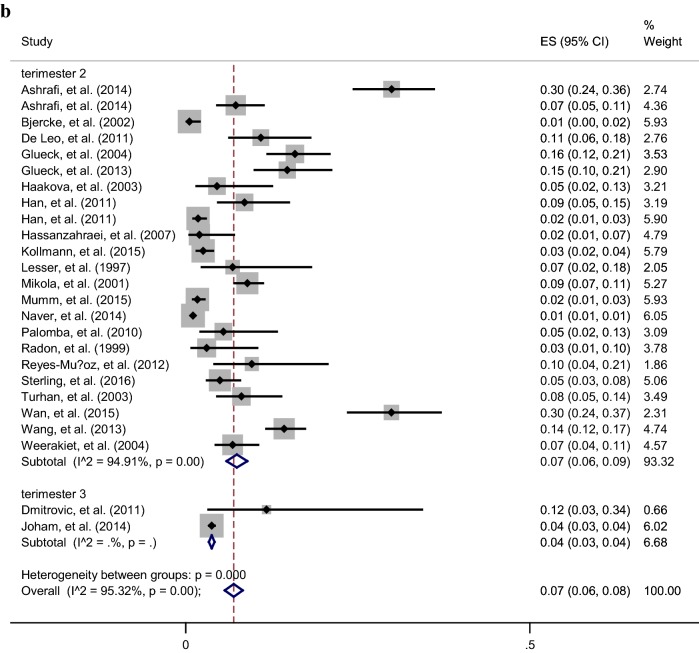
Forest plot of prevalence of GDM among women with PCOS (**a**) and healthy controls (**b**) in the first, second and third trimesters of pregnancy

The overall pooled prevalence (95% CI) of GDM among different groups was presented in Table 2. According to the Chi square test and Begg’s test, a significant heterogeneity but no publication bias was found between the studies in various subgroups. Overall, the women with PCOS were younger [Random-pooled mean (95% CI) 29.4 (28.6, 30.3) vs. 30.6 (29.7, 30.9)] and had higher BMI [Random-pooled mean (95% CI): 28.0 (26.8, 29.3) *vs.* 24.4 (23.2, 25.6)] compared with healthy controls. The Random-pooled overall prevalence of GDM among women with PCOS and healthy controls in the second trimester of pregnancy were (Random-pooled overall p = 0.19, 95% CI 0.16–0.22) and (Random-pooled overall p = 0.07, 95% CI 0.06–0.09), respectively (Fig. 1a, b).

**Table 2:** Results of heterogeneity and publication bias estimation and subgroup meta-analysis for various study population and metformin treatment among women with PCOS and without PCOS

	Sample size of participants	Chi square (*df*)	P value	Begg’s test	Pooled overall prevalence (95% CI)
Gestational diabetes in first trimester of pregnancy					
PCOS	337	4.25 (5)	0.510	0.452	0.16 (0.12, 0.19)
Without metformin therapy	257	− (1)	–	0.317	0.15 (0.10, 0.19)
Metformin therapy just before conception	44	− (1)	–	1	0.27 (0.14, 0.40)
Metformin therapy before conception till end of pregnancy	36	− (1)	–	0.317	0.13 (0.02, 0.25)
Non-PCOS	0	–	–	–	–
Gestational diabetes in second trimester of pregnancy					
PCOS	5156	1123 (58)	0.001	0.789	0.19 (0.16, 0.22)
Without metformin therapy	3008	772 (28)	0.001	0.341	0.20 (0.15, 0.25)
Metformin therapy just before conception	1232	165 (15)	0.001	0.786	0.23 (0.16, 0.30)
Metformin therapy before conception till end of pregnancy	916	61 (13)	0.001	0.555	0.11 (0.07, 0.16)
Non-PCOS	12,059	433 (22)	0.001	0.321	0.07 (0.06, 0.09)
Gestational diabetes in third trimester of pregnancy					
PCOS	575	11 (5)	0.001	0.573	0.16 (0.08, 0.23)
Without metformin therapy	495	− (1)	–	1	0.12 (0.09, 0.15)
Metformin therapy just before conception	44	− (1)	–	1	0.09 (0.01, 0.18)
Metformin therapy before conception till end of pregnancy	36	− (1)	–	1	0.22 (0.09, 0.36)
Non-PCOS	8151	− (1)	–	0.317	0.04 (0.03, 0.04)

*PCOS* polycystic ovary syndrome

### Results of meta-regression analysis

The results of univariate, and multiple weighted, linear meta-regression analysis were presented in Table 3. Unadjusted meta-regression revealed that regardless of metformin therapy, the prevalence of GDM diagnosed in second trimester among women with PCOS was 9% higher than healthy controls (β = 0.09, 95% CI 0.04, 0.16; p = 0.002) (Table 3 model 1 and Additional file 1: Fig. S2); those higher rate remained significant after adjustment of age, BMI, study design, PCOS criteria, GDM definition and quality assessment (Table 3, Models 2–8). In all studies (observational and trials), the increased risk of GDM among women with PCOS, compared to healthy controls, disappeared after the adjustment of metformin-therapy (β = 0.08, 95% CI 0.04, 0.2; p = 0.624); meta-regression analyses demonstrating that the prevalence of GDM among the women with PCOS treated before conception was statistically higher than the healthy controls (β = 0.13, 95% CI 0.06, 0.2; p = 0.001). Nevertheless, this prevalence among women with PCOS all throughout the pregnancy were as the same as the healthy controls (β = 0.037, 95% CI − 0.03, 0.1; p = 0.276) (Table 4).

**Table 3:** Meta-regression results for univariate and multiple (adjusted effect) models assessing the effect of PCOS on gestational diabetes in different trimester of pregnancy

	Trimester 1 (n = 6)		Trimester 2 (n = 82)		Trimester 3 (n = 8)	
	β (95% CI for β)	P value	β (95% CI for β)	P value	β (95% CI for β)	P value
Unadjusted model 1						
Effect PCOS	–^a^		*0.097 (0.04, 0.16)*	*0.002*	0.09 (− 0.08, 0.26)	0.234
Adjusted Models						
Model 2						
Effect of BMI	0.016 (− 0.05, 0.09)	0.522	− 0.007 (− 0.02, 0.002)	0.127	0.03 (− 0.03, 0.09)	0.359
Effect of PCOS	–^a^		*0.10 (0.05, 0.2)*	*0.001*	− 0.02 (− 0.4, 0.3)	0.887
Model 3						
Effect of BMI	0.017 (− 0.056, 0.09)	0.522	− 0.005 (− 0.02, 0.004)	0.242	0.03 (− 0.06, 0.1)	0.413
Effect of PCOS	–^a^		*0.10 (0.04, 0.2)*	*0.002*	− 0.02 (− 0.5, 0.5)	0.887
Effect of age	− 0.04 (− 0.29, 0.21)	0.650	− 0.003 (− 0.02, 0.01)	0.621	0.01 (− 0.2, 0.1)	0.413
Model 4						
Effect of BMI	0.022 (− 0.11, 0.16)	0.548	− 0.005 (− 0.02, 0.006)	0.344	0.03 (− 0.09, 0.2)	0.450
Effect of PCOS	–^a^		0.08 (0.04, 0.2)	0.624	− 0.04 (− 0.7, 0.6)	0.852
Effect of age	− 0.072 (− 0.65, 0.50)	0.647	− 0.003 (− 0.02, 0.01)	0.624	− 0.02 (− 0.4, 0.3)	0.862
Effect of metformin therapy	− 0.023 (− 0.38, 0.33)	0.803	− 0.0001 (− 0.05, 0.05)	0.997	− 0.05 (− 0.4, 0.3)	0.680
Model 5						
Effect of BMI	0.005 (− 0.1, 0.1)	0.854	− 0.004 (− 0.01, 0.01)	0.425	0.03 (− 0.03, 0.08)	0.253
Effect of PCOS	–^a^		*0.10 (0.05, 0.2)*	*0.001*	− 0.01 (− 0.2, 2)	0.840
Effect of age	0.04 (− 0.4, 0.5)	0.740	− 0.001 (− 0.01, 0.01)	0.771	− 0.1 (− 0.3, 0.06)	0.134
Effect of study design	− 0.04 (− 0.2, 0.1)	0.396	*0.05 (0.02, 0.08)*	*0.004*	0.1 (− 0.02, 0.2)	0.073
Model 6						
Effect of BMI	0.04 (− 0.1, 0.2)	0.334	− 0.005 (− 0.02, 0.004)	0.259	0.02 (− 0.07, 0.1)	0.403
Effect of PCOS	–^a^		*0.10 (0.02, 0.2)*	*0.011*	–^a^	
Effect of age	− 0.1 (− 0.6, 0.3)	0.380	− 0.004 (− 0.02, 0.01)	0.519	− 0.10 (− 0.1, 0.2)	0.761
Effect of PCOS definition	0.2 (− 0.5, 0.8)	0.396	− 0.04 (− 0.1, 0.03)	0.267	0.4 (− 0.29, 0.99)	0.144
Model 7						
Effect of BMI	0.02 (− 0.05, 0.09)	0.522	− 0.005 (− 0.02, 0.004)	0.256	0.02 (− 0.1, 0.2)	0.649
Effect of PCOS	–^a^		*0.10 (0.05, 0.2)*	*0.001*	0.02 (− 0.9, 0.9)	0.953
Effect of age	− 0.04 (− 0.3, 0.2)	0.634	− 0.002 (− 0.02, 0.01)	0.711	0.03 (− 0.3, 0.4)	0.792
Effect of quality assessment	–^a^		0.02 (− 0.05, 0.08)	0.612	− 0.06 (− 0.7, 0.6)	0.775
Model 8						
Effect of BMI	− 0.005 (− 0.12, 0.111)	0.880	− 0.006 (− 0.02, 0.004)	0.236	0.06 (− 0.1, 0.2)	0.306
Effect of PCOS	–^a^		*0.1 (0.04, 0.2)*	*0.002*	− 0.2 (− 0.9, 0.6)	0.565
Effect of age	− 0.06 (− 0.4, 0.3)	0.513	− 0.003 (− 0.02, 0.01)	0.680	− 0.02 (− 0.3, 0.2)	0.857
Effect of GDM definition	0.2 (− 0.4, 0.7)	0.352	0.01 (− 0.04, 0.06)	0.608	− 0.1 (− 0.5, 0.3)	0.459

Italic values indicate statistically significant results (p < 0.05)

^a^Insufficient data for analysis

Model 1: Univariate models assessing the effect of PCOS on Prevalence of GDM (Crude model)

Model 2: Multiple meta-regresion analysis effect of PCOS on Prevalence of GDM adjusted by BMI

Model 3: Multiple meta-regresion analysis effect of PCOS on Prevalence of GDM adjusted by BMI and age

Model 4: Multiple meta-regresion analysis effect of PCOS on Prevalence of GDM adjusted by BMI, age and metformin therapy

Model 5: Multiple meta-regresion analysis effect of PCOS on Prevalence of GDM adjusted by BMI, age and study design

Model 6: Multiple meta-regresion analysis effect of PCOS on Prevalence of GDM adjusted by BMI, age and PCOS definition

Model 7: Multiple meta-regresion analysis effect of PCOS on Prevalence of GDM adjusted by BMI, age and quality assessment

Model 8: Multiple meta-regresion analysis effect of PCOS on Prevalence of GDM adjusted by BMI, age and GDM definition

**Table 4:** Meta-regression results for effect of different metformin therapy strategy among subgroup of POCS women and healthy controls and study methodology

	Regression coefficient (95% CI)	P value
Comparison between PCOS and healthy controls		
Women with PCOS, No treated with metformin *vs.* healthy controls		
Non-RCTs~	0.10 (0.02, 0.17)	0.006
RCTs	–*	–
Women with PCOS, treated with metformin only before conception *vs.* healthy controls		
Non-RCTs	0.14 (0.07, 0.2)	0.000
RCTs	–*	–
Women with PCOS, treated with metformin before conception till the end of pregnancy vs. healthy controls		
Non-RCTs	0.035 (− 0.03, 0.1)	0.324
RCTs	–*	–*
Comparison between PCOS population		
Women with PCOS, treated with metformin only before conception vs. without metformin therapy		
Non-RCTs	0.08 (− 0.03, 0.2)	0.390
RCTs	–*	–
Women with PCOS, treated with metformin before conception till the end of pregnancy vs. without metformin therapy		
Non-RCTs	− 0.05 (− 0.07, 0.04)	0.602
RCTs	–*	–
Women with PCOS, treated with metformin before conception till the end of pregnancy vs. only before conception		
Non-RCTs	− 0.11 (− 0.24, 0.02)	0.097
RCTs	− 0.03 (− 0.25, 0.20)	0.757

* Insufficient data for analysis

~ Randomized clinical trial

Meta-regression analyses among women with PCOS treated with metformin before conception versus all throughout the pregnancy and non-users were presented in Table 4. It shown that by excluding observational studies, the prevalence of GDM among women with PCOS treated using metformin before conception until the end of pregnancy did not differ from those treated just before conception (β = − 0.09, 95% CI −0.2, 0.02; p = 0.092) or those without metformin therapy (β = − 0.05, 95% CI: − 0.07, 0.04; p = 0.301). In addition, the results remained unchanged after the subgroup analysis based methodology of RCTs and non-RCTs studies.

## Discussion

It is well documented that the prevalence of GDM among women with PCOS is higher that healthy controls. In addition, the debate whether metformin therapy can change the risk of developing GDM among women with PCOS is continued. Additionally, in term of prevention of GDM in PCOS women, the effect of metformin that can be used before conception versus all throughout the pregnancy has not been compared yet. Previous meta-analyses are controversial and inconclusive mostly due to different study designs, non-homogenous control groups and un-adjustment for possible confounding factors of age and BMI.

In an attempt to answer this important question, this meta-analysis was conducted using different approaches. Comparison of the prevalence of GDM among PCOS patients versus healthy controls showed that the prevalence of GDM regardless of metformin therapy was significantly higher in women with PCOS, and the increased risk disappeared after metformin therapy during pregnancy. However, as a source of bias, all included studies were observational that might be influenced by the various biases that influence interpretation of results. In the second approach, comparison of PCOS patients, either without or with metformin therapy in various times revealed that before and during pregnancy it could not decrease the prevalence of GDM in metformin treated women with POCS compared to those with PCOS who did not received metformin or were treated only before conception. The results of subgroup analysis based on RCTs and non-RCTs confirmed such findings. However, these results of clinical trials should also be interpreted with caution mainly due to the small number of trials, moderate quality mainly due to the randomization and blindness.

This meta-analysis confirmed earlier findings regarding the higher risk of GDM among the women with PCOS [10–13, 72, 73]. Some mechanisms have been suggested to explain the established predisposition of women with PCOS for developing GDM. It has been demonstrated that profound IR in PCOS due to peripheral target tissue resistance, decreased hepatic clearance, beta-cell dysfunction and increased pancreatic sensitivity [74, 75] is exacerbated through innate IR during pregnancy mainly by the secretion of some insulin-desensitizing placental adipokines and hormones including tumor necrosis factor (TNF)-α, growth hormone, cortisol and human placental lactogen [63, 76]. However, Metformin as an insulin sensitizer is widely used by infertile women with PCOS, which could have reduced ovarian androgens, luteinizing hormone and sex hormone binding globulins. In addition, it is helpful to improve hyperandrogenemia and insulin sensitivity via inhibiting hepatic glucose production, increasing peripheral glucose uptake and utilization, and decreasing insulin levels. Metformin recently has been considered a potentially effective agent during pregnancy to prevent GDM.

There are six meta-analyses on the effect of metformin on the occurrence of GDM in women with PCOS [14–19].

Three of them reported that metformin therapy throughout pregnancy decreased the risk of GDM in pregnant PCOS women [16, 18, 19]. However, they were subject of potential bias as their major limitations were different eligibility criteria for the type of included studies (interventional vs. observational) [18, 19] or selecting of a non-homogenous control groups (PCOS not treated, or both not treated PCOS and non-PCOS ones) [16, 18, 19].

Other three meta-analyses concluded that metformin did not significantly affect GDM among women with PCOS [14, 15, 17]. While these studies were performed a subgroup analysis of RCTs as the most stringent method of determining whether a cause-effect relation existed between the intervention and outcome, they missed some eligible studies [17], including epi-analysis [77], which re-evaluated the results of two former RCTs [50, 51] leading to duplication of previous data [17]. Also, they used the heterogeneous population as controls [15, 17] and misclassification of included studies [48] in subgroup analysis [14] was reported.

Moreover, the quality assessment and risk of bias evaluation did not perform in most those meta-analyses, and the effect of potential confounder of age and BMI did not assessed in most previous meta-analyses.

According to the PRISMA guidelines, the current meta-analysis has standard criteria and presents reliable results. The main strength of this meta-analysis was the large number of eligible studies reviewed in this study, and also the adjustment for potential confounders, which made it possible to present the real feature of this syndrome. In addition, using the homogenous controls (PCOS not treated, or both not treated PCOS and non-PCOS ones) helped us to control the source of heterogeneity. Moreover, the impact of time and duration of metformin therapy for optimum reduction of GDM were evaluated. In addition, most studies included an estimated moderate or high quality with the low risk of bias that helped us provide high quality evidence, sensitivity analysis based on risk of bias showed no difference as well.

Nevertheless, it should be noted that, despite this meta-analysis, it seems the evidence about the metformin therapy among women with PCOS who had risk factor for GDM e.g. advanced maternal age [78], previous macrosomia [79], maternal obesity [80], maternal impaired glucose tolerance [81], ethnicity [82] and family history of diabetes [83], is insufficient and treatment should be prescribed individually for each patient.

However, as the limitations of the present study, there was inadequate evidence, and lack of large scale well-designed RCTs to establish the influence of metformin therapy in the various trimesters of pregnancy on the prevalence of GDM. While the onset of metformin therapy before conception was exactly specified, the duration of metformin treatment before pregnancy was unclear in some included studies and we could not adjust it as a potential confounding factor in this meta-analysis. Moreover, most studies were performed in infertility treatment center, may limit the validity of the results.

## Conclusion

The main body of literature in the current meta-analysis was observational, which may be mixed with some sources of bias. Also, a lack of well-designed and high quality interventional studies means that the findings should be interpreted with cautious. In this respect, decisions regarding the continuation or discontinuation of metformin therapy in women with PCOS are somewhat arbitrary and can be made individually based on the patient’s condition given the presence or absence of other GDM risk factors. Additional well-designed RCTs still need for precise recommendation.

## Additional file

**Additional file 1.** Additional figures and tables.

## Data Availability

The datasets used and analysed during the current study are available from the corresponding author on reasonable request

## Abbreviations

GDM: gestational diabetes mellitus
PCOS: polycystic ovary syndrome
BMI: body mass index
RCT: randomized clinical trial
IR: insulin resistance
PRISMA: preferred reporting items for systematic reviews and meta-analyses
NRS: nonrandomized Studies
