# Supplementary material for: Metformin therapy before conception versus throughout the pregnancy and risk of gestational diabetes mellitus in women with polycystic ovary syndrome: a systemic review, meta-analysis and meta-regression
Source: Diabetol Metab Syndr. 2019 Jul 23;11:58. doi: 10.1186/s13098-019-0453-7 (PMC6651943; doi:10.1186/s13098-019-0453-7)
Supplement: Supplementary file 1 — Additional file 1. Additional figures and tables. [file 13098_2019_453_MOESM1_ESM.docx]

Additional file

**Additional Figure S1.** Flow chart of the literature search for the systematic review and meta-analysis.

## Screening

## Included

## Eligibility

## Identification

Records identified through database searches: (n = 1397)

(n = 2759)

Records remaining after duplicates removed: (n = 924)

Records screened based on abstract: (n =107)

Full-text articles assessed for eligibility: (n = 67)

Total number of studies groups included in meta-analysis (n=48)

**Additional Figure S2**: risk of bias in randomized clinical trial

A:

| Author, date | Bias in Random sequence generation | Bias in Allocation concealment | Bias in Blinding of participants and personnel | Bias in Blinding of outcome assessment | Bias in Incomplete outcome data | Bias in Selective outcome  reporting |
| --- | --- | --- | --- | --- | --- | --- |
| Begum, et al. (2009) 5 | **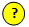** | **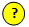** | **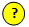** | **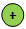** | **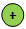** | **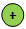** |
| Fougner, et al. (2008) 38 | 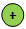 | 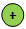 | 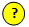 | 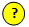 | **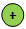** | **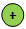** |
| Vanky, et al. (2004) 113 | 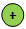 | 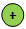 | 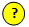 | 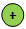 | **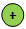** | **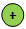** |
| Vanky, et al. (2010) 114 | 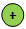 | 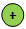 | 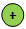 | 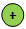 | 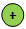 | 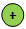 |
| 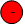 Yes (high risk of bias) 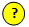 Unclear 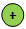 No (low risk of bias)  **RCT** | | | | | | |

B:

**Additional Figure S3**: risk of bias in n-RCT

A:

| Author, date | Bias due to confounding | Bias in selection of participations | Bias in interventions classification | Bias in deviations from intended interventions | Bias due to missing data | Bias in measurement of outcomes | Bias in selection of the  reported result |
| --- | --- | --- | --- | --- | --- | --- | --- |
| Abd El Hameed et al. (2011) 1 |  |  |  |  |  |  |  |
| No (low risk of bias) probably no Non information  Yes (High risk of bias) Probably yes | | | | | | | |

B:

**Additional Figure S4**: risk of bias in case- control study

**A:**

| Author, date | Bias in assessment of exposure | Bias in development of outcome of interest in case and controls | Bias in selection of cases | Bias in selection of controls | Bias in control of prognostic variable (without case and control matching or adjustment in statistical methods ) |
| --- | --- | --- | --- | --- | --- |
| Ashrafi, et al. (2014) 11 |  |  |  |  |  |
| Ashrafi, et al. (2017) 12 |  |  |  |  |  |
| Dmitrovic, et al. (2011) 31 |  |  |  |  |  |
| Joham, et al. (2014) 63 |  |  |  |  |  |
| Nawaz, et al. (2008) 86 |  |  |  |  |  |
| Vanky, et al. (2011) 115 |  |  |  |  |  |
| Zhang, et al. (2016) 126 |  |  |  |  |  |
| Mehrabian, et al. (2013) 127 |  |  |  |  |  |
| Definitely No (low risk of bias) probably no  Definitely yes (high risk of bias) probably Yes | | | | | |

**B:**

**Additional Figure S5**: risk of bias in cohort study

**A:**

| Author, date | Bias in selection of exposed and non‐exposed cohorts | Bias in assessment of exposure | Bias in present of outcome of interest at start of study | Bias in control of prognostic variables (with matching or adjusting) | Bias in in the assessment of the presence or absence of prognostic factors | Bias in in the assessment of outcome | Bias in adequacy about follow up of cohorts |
| --- | --- | --- | --- | --- | --- | --- | --- |
| Bjercke, et al. (2002) 17 |  |  |  |  |  |  |  |
| D’Anna, et al. (2012) 25 |  |  |  |  |  |  |  |
| De Fre`ne, et al. (2014) 26 |  |  |  |  |  |  |  |
| De Leo, et al. (2011) 27 |  |  |  |  |  |  |  |
| deWilde, et al. (2015) 29 |  |  |  |  |  |  |  |
| Elkholi, et al. (2016) 36 |  |  |  |  |  |  |  |
| Glueck, et al. (2004) 40 |  |  |  |  |  |  |  |
| Glueck, et al. (2004) 41 |  |  |  |  |  |  |  |
| Glueck, et al. (2002) 44 |  |  |  |  |  |  |  |
| Glueck, et al. (2013) 45 |  |  |  |  |  |  |  |
| Glueck, et al. (2008) 47 |  |  |  |  |  |  |  |
| Glueck, et al. (2012) 48 |  |  |  |  |  |  |  |
| Haakova, et al. (2003) 52 |  |  |  |  |  |  |  |
| Han, et al. (2011) 54 |  |  |  |  |  |  |  |
| Hassanzahraei, et al. (2007) 55 |  |  |  |  |  |  |  |
| Khattab, et al. (2011) 66 |  |  |  |  |  |  |  |
| Kollmann, et al. (2015) 69 |  |  |  |  |  |  |  |
| Lesser, et al. (1997) 75 |  |  |  |  |  |  |  |
| Mikola, et al. (2001) 83 |  |  |  |  |  |  |  |
| Mumm, et al. (2015) 84 |  |  |  |  |  |  |  |
| Naver, et al. (2014) 85 |  |  |  |  |  |  |  |
| Ott, et al. (2014) 91 |  |  |  |  |  |  |  |
| Palomba, et al. (2010) 92 |  |  |  |  |  |  |  |
| Paradisi, et al. (1998) 95 |  |  |  |  |  |  |  |
| Radon, et al. (1999) 99 |  |  |  |  |  |  |  |
| Reyes-Muñoz, et al. (2012) 100 |  |  |  |  |  |  |  |
| Sterling, et al. (2016) 105 |  |  |  |  |  |  |  |
| Turhan, et al. (2003) 109 |  |  |  |  |  |  |  |
| Vollenhoven , et al. (2000) 111 |  |  |  |  |  |  |  |
| Veltman-Verhulst, et al. (2010) 117 |  |  |  |  |  |  |  |
| Wan, et al. (2015) 119 |  |  |  |  |  |  |  |
| Wang, et al. (2013) 120 |  |  |  |  |  |  |  |
| Weerakiet, et al. (2004) 121 |  |  |  |  |  |  |  |
| Xia, et al. (2017) 124 |  |  |  |  |  |  |  |
| Definitely No (low risk of bias) probably no  Definitely yes (high risk of bias) probably Yes | | | | | | | |

B:

Additional Figure S6: Scatter bubble plots fitted association line for prevalence of gestational diabetes and polycystic ovary syndrome status at second (A) and third (B) trimester of pregnancy, regardless of metformin therapy

**Additional Table S1. Quality assessment of included studies using the Consort Assessment Scale for interventional studies**

| Author | Methods | | | | | | | | | | | | | | | | |
| --- | --- | --- | --- | --- | --- | --- | --- | --- | --- | --- | --- | --- | --- | --- | --- | --- | --- |
|  | Trial design | | Participants | | Interventions | Outcomes | | Sample size | | Randomization | | Allocation concealment mechanism | Implementation | Blinding | | Statistical methods | |
|  | a | b | a | b |  | a | b | a | b | a | b |  |  | a | b | a | b |
| Abd El Hameed, et al. (2011) | + | - | + | + | + | + | - | - | - | - | - | - | - | - | - | + | - |
| Begum, et al. (2009) | - | - | + | + | + | - | - | - | - | - | - | - | - | - | - | + | + |
| Fougner, et al. (2008) | - | - | + | + | + | + | - | - | - | + | + | - | - | - | + | + | + |
| Vanky, et al. (2004) | - | - | + | + | + | + | - | - | - | - | + | - | - | + | + | + | + |
| Vanky, et al. (2010) | - | - | + | + | + | + | - | + | - | - | + | - | - | + | + | + | + |

| Author | Results | | | | | | | | | | Total | quality |
| --- | --- | --- | --- | --- | --- | --- | --- | --- | --- | --- | --- | --- |
|  | Participant flow (a diagram is strongly recommended) | | Recruitment | | Baseline data | Numbers analyzed | Outcomes and estimation | | Ancillary analyses | Harms |  |  |
|  | a | b | a | b |  |  | a | b |  |  |  |  |
| Abd El Hameed, et al. (2011) | + | - | + | - | + | + | + | - | - | - | 12 | moderate |
| Begum, et al. (2009) | + | - | - | - | + | + | + | - | - | - | 9 | moderate |
| Fougner, et al. (2008) | + | - | - | - | + | + | + | - | - | - | 11 | moderate |
| Vanky, et al. (2004) | + | - | + | - | + | + | + | - | - | - | 14 | moderate |
| Vanky, et al. (2010) | + | + | + | - | + | + | + | + | + | - | 17 | moderate |

**Additional Table S2.** Quality assessment of included studies using the Newcastle–Ottawa Quality Assessment Scale for cross-sectional studies

| **Author** | **SELECTION** | | | | **COMPARABILITY** | | **Outcome** | | **Total scores** | **Quality** |
| --- | --- | --- | --- | --- | --- | --- | --- | --- | --- | --- |
|  | Representativeness of the samples | Sample size | Non-responders | Ascertainment of the exposure (risk factor) | A: study controls for age and/or BMI | B: control for any additional factor | Assessment of the outcome  a) Independent blind assessment ++  b) Record linkage ++  c) Self report + | Statistical test |  |  |
| Ashrafi,  et al. (2014) | - | + | - | + | + | + | ++ | + | 7 | high |
| Ashrafi,  et al. (2017) | + | - | - | - | - | - | ++ | + | 4 | moderate |
| Dmitrovic,  et al. (2011) | + | - | - | + | + | - | + | + | 5 | moderate |
| Elkholi,  et al. (2016) | - | - | + | + | - | - | + | + | 5 | moderate |
| Joham,  et al. (2014) | + | - | + | + | + | + | ++ | + | 8 | high |
| Vanky, et al. (2011) | + | - | - | + | + | - | ++ | + | 6 | high |
| Zhang, et al. (2016) | - | - | + | + | - | - | ++ | + | 5 | moderate |

**Additional Table S3.** Quality assessment of included studies using the Newcastle–Ottawa Quality Assessment Scale for cohort studies

|  | **SELECTION** | | | | **COMPARABILITY** | | **Outcome** | | | **Total scores** | **Quality** |
| --- | --- | --- | --- | --- | --- | --- | --- | --- | --- | --- | --- |
| **Author** | Representativeness of the exposed cohort | Selection of the non-exposed cohort | Ascertainment of exposure | No outcome of interest at start of study | A: Study controls for age and/or BMI | B: Study controls for other confounders | A: Independent blind assessment  B: Record linkage | follow-up long enough for outcome | A:complete of follow up of cohorts B: lost to follow up less than 20% |  |  |
| Bjercke, et al. (2002) | - | + | - | + | - | - | + | + | + | 5 | moderate |
| D’Anna,  etal.(2012) | - | - | - | + | + | + | + | + | + | 5 | moderate |
| De Fre`ne,  et al.(2014) | - | + | - | + | + | + | + | + | + | 7 | moderate |
| De Leo,  et al.(2011) | - | + | - | + | - | - | + | + | + | 5 | moderate |
| deWilde,  et al.(2015) | - | - | + | + | - | - | + | + | + | 5 | moderate |
| deWilde,  et al.(2014) | - | - | + | + | + | + | + | + | + | 7 | high |
| Glueck,  et al.(2004) | - | - | + | + | - | - | + | + | + | 5 | moderate |
| Glueck,  et al.(2004) | - | - | + | + | - | - | + | + | + | 5 | moderate |
| Glueck,  et al.(2002) | - | - | + | + | - | - | + | + | + | 5 | moderate |
| Glueck,  et al.(2013) | - | + | + | + | - | - | - | + | + | 5 | moderate |
| Glueck,  et al.(2008) | - | - | + | + | - | - | + | + | + | 5 | moderate |
| Glueck,  et al.(2002) | - | - | + | + | - | - | + | + | + | 5 | moderate |
| Haakova,  et al.(2003) | - | - | + | + | + | - | + | + | + | 6 | high |
| Han,  et al.(2011) | + | - | - | - | - | - | - | + | + | 4 | moderate |
| Hassanzahraeiet al. (2007) | - | - | + | + | + | - | + | + | + | 6 | high |
| Khattab,  et al.(2011) | - | - | + | + | - | - | + | + | + | 5 | moderate |
| Kollmann,  et al.(2015) | + | + | + | + | + | - | + | + | + | 6 | high |
| Lesser,  et al.(1997) | - | - | + | + | + | - | + | + | + | 6 | high |
| Mehrabian,  et al.(2013) | - | - | + | + | - | - | + | + | + | 5 | moderate |
| Mikola,  et al.(2001) | - | + | + | + | - | + | + | + | + | 7 | high |
| Mumm,  et al.(2015) | - | - | + | + | + | + | + | + | + | 8 | high |
| Naver,  et al.(2014) | - | + | + | + | + | + | - | + | + | 7 | high |
| Nawaz,  et al.(2008) | - | - | - | + | + | + | + | + | + | 6 | high |
| Ott,  et al.(2014) | - | - | + | + | + | - | + | + | + | 6 | high |
| Palomba,  et al.(2010) | - | - | + | + | - | - | + | + | + | 5 | moderate |
| Paradisi,  et al.(1998) | - | - | + | + | - | - | + | + | + | 5 | moderate |
| Radon,  et al.(1999) | - | - | + | + | + | - | + | + | + | 6 | high |
| Reyes-Muñoz, et al. (2012) | - | - | + | + | + | + | + | + | + | 7 | high |
| Sterling,  et al.(2016) | - | - | + | + | + | + | + | + | + | 5 | high |
| Turhan,  et al.(2003) | - | - | + | + | + | - | + | + | + | 6 | high |
| Vollenhoven , et al. (2000) | - | - | + | + | + | + | + | + | + | 7 | high |
| Veltman-Verhulst,  et al.(2010) | - | - | + | + | + | + | + | + | + | 7 | high |
| Wan, et al. (2015) | - | - | + | + | + | - | + | + | + | 6 | high |
| Wang, et al. (2013) | - | - | + | + | + | - | + | + | + | 6 | high |
| Weerakiet,  et al.(2004) | - | - | + | + | + | - | + | + | + | 6 | high |
| Xia, et al. (2017) | - | - | + | + | + | - | + | + | + | 6 | high |
